# Genetic Basis of Hypertrophic Cardiomyopathy in Cats

**DOI:** 10.3390/cimb46080517

**Published:** 2024-08-12

**Authors:** Arkadiusz Grzeczka, Szymon Graczyk, Robert Pasławski, Urszula Pasławska

**Affiliations:** 1 Department for Basic and Preclinical Sciences, Institute of Veterinary Medicine, Faculty of Biological and Veterinary Sciences, Nicolaus Copernicus University in Torun, 87-100 Torun, Poland; grzeczka@umk.pl (A.G.); graczyk72@gmail.com (S.G.); 2 Department of Veterinary Surgery, Institute of Veterinary Medicine, Faculty of Biological and Veterinary Sciences, Nicolaus Copernicus University in Torun, 87-100 Torun, Poland; r.paslawski@umk.pl; 3 Department of Diagnostics and Clinical Sciences, Institute of Veterinary Medicine, Faculty of Biological and Veterinary Sciences, Nicolaus Copernicus University in Torun, 87-100 Torun, Poland

**Keywords:** MYBPC3, ALMS1, TNNT2, MYH7, HCM, Maine Coon, Ragdoll, epigenetic

## Abstract

Hypertrophic cardiomyopathy (HCM) is a common cardiovascular condition in cats, affecting yth males and females of all ages. Some breeds, such as Ragdolls and Maine Coons, can develop HCM at a young age. The disease has a wide range of progression and severity, characterized by various pathological changes in the heart, including arteritis, fibrous tissue deposition, and myocardial cell hypertrophy. Left ventricular hypertrophy, which can restrict blood flow, is a common feature of HCM. The disease may persist into old age and eventually lead to heart failure and increased diastolic pressure. The basis of HCM in cats is thought to be genetic, although the exact mechanisms are not fully understood. Mutations in sarcomeric proteins, in particular myosin-binding protein C (MYBPC3), have been identified in cats with HCM. Two specific mutations, MYBPC3 [R818W] and MYBPC3 [A31P], have been classified as ‘pathogenic’. Other variants in genes such as MYBPC3, TNNT2, ALMS1, and MYH7 are also associated with HCM. However, there are cases where cats without known genetic mutations still develop HCM, suggesting the presence of unknown genetic factors contributing to the disease. This work aims to summarise the new knowledge of HCM in cats and the alterations in cardiac tissue as a result of genetic variants.

## 1. Introduction

Hypertrophic cardiomyopathy (HCM) is the most common cardiovascular condition seen in cats. HCM affects individuals of both sexes (with males being slightly more predisposed) and of all ages [1,2]. In breeds such as the Ragdoll and Maine Coon, HCM manifests itself extremely rapidly and is diagnosed as early as a few months of age [3,4,5,6,7,8]. The disease shows a high heterogeneity in terms of progression and the severity of the lesions [9]. The affected heart is characterized by intramural atherosclerosis of the coronary arteries, fibrous tissue deposition in the left ventricular wall, myocyte hypertrophy characterized by thick muscle fibers with oval and vesicular nuclei with prominent nucleoli, myocardial replacement fibrosis (replacement of dead cardiomyocytes), myocardial interstitial fibrosis and endocardial fibrosis [10]. In addition, diagnostic capabilities for determining the extent of myocardial decay have been obtained using micro-CT [11]. Left ventricular myocardial hypertrophy may be symmetrical or asymmetrical and may lead to outflow tract restriction [12,13,14]. The subclinical stage of the disease may persist into old age [9]. However, progressive myocardial fibrosis leads to worsening myocardial relaxation dysfunction, resulting in increased diastolic pressures [1,15]. Consequently, the occurrence of increasing left atrium (LA) pressures enlarges the size of the LA, predisposing it to the formation of embolic material and the risk of feline arterial thromboembolism (FATE) [1]. Furthermore, because of HCM, heart failure develops, or sudden cardiac death occurs [16]. Continuous patient monitoring and screening of asymptomatic cats is crucial in controlling the disease [17]. Echocardiography is best for assessing the incidence and progression of HCM. In addition, biomarkers are available to aid in diagnoses, such as N-terminal prohormone of brain natriuretic peptide (NT-proBNP), cardiac troponin (cTn), and collagen type 1 metabolite [18,19,20,21]. Although the phenotype of HCM is well known, a definitive cause for the development of HCM in cats is still lacking. Although HCM is known to be a genetic disease, defining the trajectory of HCM development is difficult [22]. All mutations involve sarcomeric proteins (except one report from Preprints Springer, where the authors first reported an HCM-associated intron mutation of the Cysteine and Glycine Rich Protein gene (CSRP3) [23]). We now know that there are two genetic variants (MYBPC3:c.2453C > T [R818W] and MYBPC3:c.91G > C [A31P]) segregating races that are associated with the development of HCM in cats [24,25]. Both involve the myosin-binding protein C (MYBPC3) [24,25]. In contrast, four other variants (MYBPC3:c.220G > A [A74T], TNNT2:c.95-108G > A, ALMS1:c.7384G > C [G2462R], MYH7:c.5647G > A [E1883K]), responsible for the heavy chain, thin filaments and myosin-binding C protein, are suspected to be possibly associated with HCM. In addition, hundreds of SNPs in key genes have been identified by DNA sequencing of diseased cats, but for the time being, we are unable to assign significance to them [26,27,28,29,30,31]. According to the recently used classification of the American College of Medical Genetics and Genomics, two mutations (MYBPC3:c.2453C > T [R818W] and MYBPC3:c.91G > C [A31P]) were considered ‘pathogenic’ [32]. MYH7:c.5647G > A [E1883K] was classified as ‘probably pathogenic’, ‘probably benign’ is ALMS1:c.7384G > C [G2462R], TNNT2:c.95-108G > A. In contrast, a mutation of unknown significance was considered MYBPC3:c.220G > A [A74T] [32]. To date, most is known about the genetic background of HCM in Maine Coon, Ragdoll, and Sphynx cats [24,25,26,33,34,35]. Cats may be homozygous or heterozygous for a particular mutation variant, with which the rate of progression and the nature of the lesions will be associated. However, there are individuals of breeds predisposed to HCM who have not been genetically affected and yet develop severe forms of HCM [28]. Furthermore, there is still a significant proportion of HCM patients who are cats without a specific breed and often do not have any of the known and suspected variants. Most likely, there are still unknown mutations that contribute to the development of HCM. Therefore, data from the full range of mRNA molecules expressed by the body as a result of the stressful effects of ‘toxic proteins’ embedded in the sarcomeric structure and proteomic studies that reveal peptidome profiles in cats with the genetic HCM variants present are valuable [36,37].

This literature review aims to summarise the knowledge of the genetic basis of HCM development, which has been greatly expanded in recent years.

## 2. Gene Mutations

### 2.1. MYBPC3 Protein Mutation

The MYBPC3 gene is located on chromosome D1 and consists of 35 exons (Gene ID: 101094698). When transcribed, the gene yields myosin-binding protein C (MYBPC3). MYBPC3 is similar to MYBPC1 and MYBPC2, two other proteins found in muscle tissue. However, they are subject to additional expression in skeletal muscle tissue, unlike MYBPC3 [38,39]. The feline MYBPC3 protein is structured similarly to human MYBPC3 and consists of 11 subunits, of which C0 is specific for the cardiac type of MYBCP3. The other domains are C1, C2, C3, C4, C5, C8, C10 and, together with C0, are immunoglobulin-like domains. In contrast, the C6, C7, and C9 domains form the fibronectin type III region [40]. The N-terminal and C-terminal domains differ dramatically in structure. Closer to the N-terminal domain, between C1–C2, an accumulation of phosphorylation sites is located, while the C0–C1 region is able to associate with myosin and actin [41,42]. The C-terminal domain, on the other hand, is fibronectin-like and has the potential to associate with the myosin-heavy chain. MYBPC3 is located in a transverse band in the A band of the sarcomere and attaches to titin and myosin heavy chain B [43]. MYBPC3 is crucial for the structure and organization of sarcomeres and the maintenance of normal cardiac function [44]. Primarily, it regulates myofilament Ca^2+^ sensitivity, contractile force, and diastolic function of the heart. In mouse knockout models, eliminating the effect of MYBPC3 results in myocardial hypertrophy, interstitial fibrosis, increased force of contraction, increased Ca^2+^ sensitivity, and decreased peak relaxation [45]. Thus, the correct function of the functional C- and N-terminal domains of the protein, which limit the number of cross-bridges formed (regulating the force of contraction), appears to be crucial, and the presence of MYBPC3 is crucial for full myocardial relaxation [46]. Similar molecular conditions for the development of HCM have been suggested in cats [17]. What is puzzling, however, is the dramatic difference between human HCM and the feline model in terms of the mutations present. Indeed, MYBPC3 has been reported to be mutated in three ways in human medicine: frame shifts, missense, and splicing variants [47], among which frame shifts predominate [48]. In contrast, only missense variants have so far been reported in cats [24,25,27]. Mutations based on frame shifts contribute to a change in DNA sequence and the synthesis of shorter proteins and consequently cause mRNA degradation and reduction of MYBPC3 in cardiac tissue [49]. Missense mutations result in mutant proteins by changing single nucleotide pairs without altering the length of their DNA sequence, leading to stable mutant proteins [50,51]. It can, therefore, be assumed that missense mutations have different pathogenetic mechanisms, each of which is unique to the mutant protein. However, this does not exclude common molecular pathways. In human medicine, it has been recognized that all MYBPC3 variants cause the same abnormality based on similar pathways. This is based on the fact that the HCM phenotype is the same, regardless of the mutation type and MYBPC3 variant [40]. The different mutation variants discovered in cats also lead to similar phenotypes and, therefore, may also trigger similar mechanisms. MYBPC3:c.91G > C [A31P] was occluded earliest and involved a mutation in exon 3, where guanine was changed to cytosine in codon 31 in affected Maine Coon cats [24] and is autosomal dominant inheritance [52]. As a consequence of the change of one base pair, G to C, one of the amino acids, alanine, was changed to proline [24]. Replacing alanine with trans-proline changes the polarity of the N-terminal domain from −1847 kJ/mol to +431 kJ/mol [53]. In addition, proline alone is less able to form hydrogen bonds, resulting in one less bond in the molecule compared to the MYBPC3 protein with alanine [53]. It also has strong folding properties [54,55] and, thus, can change the conformation of the protein or alter the accessibility of other parts of the protein, as is the case with tryptophan at codon 42 [53]. Despite the altered structure, the protein is incorporated into the sarcomere structure [56]. This mutation may alter the potential of MYBPC3 to efficiently bind to actin [53], as it is located in the connecting region between C0 and C1 [24]. However, the mutation did not affect the level of protein phosphorylation or TnI phosphorylation [57]. Therefore, the impact of the MYBPC3:c.91G > C [A31P] variant can be summarised as a destabilization of key residues involved in the interaction with actin and an effect on actin binding by altering intramolecular interactions and altering surface electrostatic potentials. 

A further variant was discovered during a study of familial hypertrophic cardiomyopathy, detected in parents and three Ragdoll offspring [25]. Importantly, the new mutation was the only DNA sequence change, i.e., A31P was not present. In the case of this family, the exchange was again guanine, but this time to thymine. In all cats, this change occurred at codon 818 (exon 26), and consequently, tryptophan was created instead of arginine [25], and the mode of inheritance is similar to the variant found in Maine Coon, i.e., autosomal dominant [25] (Table 1). A similar mutation was subsequently discovered in humans [58]. The MYBPC3:c.2453C > T [R818W] variant is located in domain 6, a type III fibronectin region, which may disrupt the binding of MYBPC3 to the myosin heavy chain [25]. For MYBPC3:c.91G > C [A31P] and MYBPC3:c.2453C > T [R818W], it has been shown that a cat with HCM has 1.5-2.0 times higher Ca^2+^ sensitivity than the troponin of a healthy cat [57]. The MYBPC3:c.220G > A [A74T] variant was detected in exon 2. The encoding of mutant proteins and their incorporation into the sarcomere leads to severe dysfunction of the basic building block of the heart. This effect is referred to as ‘toxic proteins’. To prevent this, protein quality monitoring is activated, in which the ubiquitin-proteasome system plays a key role [59]. In addition, preventive processes degrade the mRNA of misfolded proteins. However, these limitations lead to haploinsufficiency, a state in which a deficiency exists. In human HCM, haploinsufficiency is recognized as a major cause of its development on the background of truncating mutations. A protein resulting from a missense mutation will more easily bypass quality control processes, as has been demonstrated in humans [60]. Furthermore, in cats, reduced amounts of cMyBP-C, myosin, titin, and cardiac actin were detected. A 69% reduction in cMYBPC was detected for the heterozygous condition and 88% for the homozygous condition [24]. Instead, a 1.25- to 3-fold increase in mRNA was detected [24]. However, this was not confirmed in subsequent studies where haploinsufficiency did not occur [53]. In a recent study, haploinsufficiency was again not detected in the whole group, but protein levels were reduced by approximately 20–30% in several cats [57]. Interestingly, haploinsufficiency was not detected in this study in a cat that was homozygous for MYBPC3:c.2453C > T [R818W] [57]. Thus, as can be seen, the similarity of BYBPC3 mutations in feline and human HCM is high, but more studies on this model are needed to discover the truer impact of individual mutations.

### 2.2. Myosin Heavy Chain Mutation

Myosin heavy chains are large proteins composed of two elements: a head and a tail [61,62]. The chains are formed from two isoforms, MYH6 and MYH7. However, MYH7 is crucial for muscle function in adults. The myosin heavy chain 7 (MYH7) variant is located on chromosome B3, which consists of 40 exons (Gene ID: 101096736) [30]. Mutations in this gene were detected in a hybrid cat [30]. The MYH7 c.5647G > A [E1883K] variant is most likely to be autosomal dominant inheritance [30]. The mutation changes glutamic acid to lysine, thereby altering the actions of the binding competence domain (ACD). As indicated by the authors of the discovery, the mutation may alter a specific fragment of the C-terminal domain responsible for anchoring the thick fibers of the sarcomere [30]. The location of the mutation suggests that it may similarly affect sarcomere function as the MYBPC3:c.2453C > T [R818W] mutation. However, it is a very rare variant with a likely pathogenic impact [32]. Not yet published data (Preprints Springer) also report another novel intron heavy chain mutation that affects the MYH7 gene (MYH7 (B3:76168426 G > A)) and was detected in Burmese cats [23].

### 2.3. Alstrom’s Syndrom

A mutation in the ALMS1 gene has been discovered in familial HCM Sphynx. The ALMS1:c.7384G > C [G2462R] variant is located in chromosome A3 in exon 12 (Gene ID: 101098372) [33]. The ALMS1:c.7384G > C [G2462R] variant changes the amino acid from glycine, a non-polar amino acid, to positively charged arginine [33]. Alstrom syndrome was originally diagnosed in humans [63] and is responsible for multisystem impairment and is characterized by retinal pigmentary degeneration, hearing loss, obesity, diabetes, nephropathy, and occasionally cardiomyopathy [63]. In cats, recent screening has revealed five new ALMS1 variants at different locations [35]. However, their close association with HCM has still not been indicated. Even in humans, cardiomyopathies on the background of Alstrom syndrome are rare, and the mechanism of its development is not fully understood. It is suspected that they may result from impaired proliferation processes, as evidenced by high Ki-67 protein activity in both human and feline HCM on an ALMS1 background [33,64]. High levels of Ki-67 protein are noted among stimulated (proliferating) cells, whereas it is absent in the resting phase of the G0 cell cycle [65]. In the case of the Sphynx family studied, affected cats had 10-fold higher Ki-67 activity than control cats without HCM [33].

### 2.4. Thin Filaments Mutations

Cardiac troponin T (cTnT) is one of the key proteins embedded in the thin filaments that bind to tropomyosin. In addition, it is responsible for regulating the response to changes in intracellular ion concentration. The mutant proteins arise from an intron mutation in the TNNT2 gene (TNNT2:c.95-108G > A) [66]. This is the only variant that is located in an intron in the F1 gene (Gene ID: 493940). The TNNT2 gene was first identified as a candidate gene in the British Shorthair Cat, Sphynx, Maine Coon, and Siberian Cat [26]. However, the identified SNPs have not been associated with HCM [26]. In a subsequent study, the reported TNNT2:c.95-108G > A variant in the Maine Coon was associated with HCM [66]. However, a recent report did not confirm this [29]. Furthermore, extensive studies have revealed that it is also prevalent among predisposed breeds (including Maine Coon and Ragdoll) [32]. Increased Ca^2+^ sensitivity in cats with HCM has been reported in cats with MYBPC3:c.91G > C [A31P] and MYBPC3:c.2453C > T [R818W] mutations [57]. However, in the year of the study, the TNNT2 variant was not yet known, which does not exclude the possibility of the presence of TNNT2 mutations. Moreover, CA^2+^ dysregulation, which is normally mediated by cardiac troponin T, was indicated [57]. Furthermore, it has been shown that this mutation may be responsible for splicing disruption in exon 3 [66]. Mutations in exon 3 lead to disruption of the splicing of the N-terminal domain to actin, which may reveal the dual effect of the TNNT2:c.95-108G > A mutation [53].

### 2.5. Occurrence of Genetic Variants

**Figure 1:**
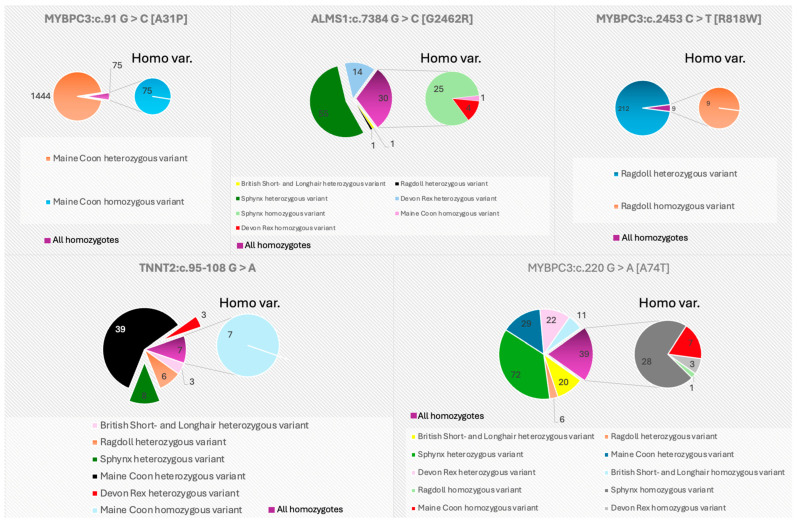
Presence of genes suspected to be associated with HCM in different cat breeds. The large circle represents all the variants recorded by Boeykens et al. [32], and the small circle is a breakdown of the homozygotes that occurred in each breed. For the graphs, data from a recent allele frequency study of six variants associated with hypertrophic cardiomyopathy in cats were used, which can be related to the actual occurrence of these variants in cats. Adapted with permission from Ref. [32].

The individual variants are breed-specific and are only incidentally detected in other breeds or hybrids (Figure 1). Furthermore, it has been suggested that the presence of the MYBPC3:c.91G > C [A31P] mutation in cats other than the Maine Coon is the result of crossbreeding with this breed of other individuals. Similarly, the occurrence of MYBPC3:c.2453C > T [R818W] in cats other than Ragdolls is associated with crossbreeding or the occurrence of Ragdolls in the formation of the breed. According to recent reports, the frequency of these genes has decreased significantly [32]. The prevalence of MYBPC3:c.91G > C [A31P] among Maine Coon individuals has decreased over the years from tens of percent [27,67,68,69] to 6% in a recent study [32]. In contrast, MYBPC3:c.2453C > T [R818W] decreased from 27% [69] to 2% [32]. Among cats with HCM, 66.1% were WT, 28.8% were heterozygotes and 5.1% were homozygotes [70]. The diametrical decrease in the frequency of these two genes is the result of increased testing for the variant and exclusion of carriers from reproduction. However, breeding utility has not been acquired by testing for the MYBPC3:c.220G > A [A74T] variant. Interestingly, commercial testing appeared earlier than the first scientific reports of a possible association of MYBPC3:c.220G > A [A74T] with the development of HCM [27]. The several identifications of this variant as a widespread missense mutation with no association with any phenotype probably reduced the interest of breeders in this gene [27,68]. Therefore, its prevalence has remained virtually unchanged over the years [27,32,68]. A similar level of prevalence was maintained by TNNT2:c.95-108G > A, occurring in about 20–30% of the populations studied [29,32]. In contrast, MYH7:c.5647G > A [E1883K], as previously mentioned, remains noted only once [30]. In contrast, the variant detected in the ALMS1 gene is most prevalent in many cat breeds, among which Sphynx is predominant [32,33,34].

**Table 1:** Prevalence of genetic variants associated with HCM ever identified in individual breeds. Bold indicates the breed in which the variant was first detected.

MYBPC3:c.91 G > C [A31P]	**Maine coon**	[71]
	Pixiebob longhair	
	Siberian	
	No-breed	
	Ragdoll	[32]
	Munchkin	[72]
	Scottish fold	
MYBPC3:c.2453 C > T [R818W]	American bobtail longhair	[32,71]
	American bobtail shorthair	
	Highlander	
	Munchkin	
	RagaMuffin	
	No-breed	
	**Ragdoll**	
MYBPC3:c.220 G > A [A74T]	British shorthair	[32]
	British longhair	
	Ragdoll	
	Sphynx	
	**Maine coon**	
	Devon rex	
	Norwegian forest cats	[68]
	Persian	
	Bengalskich	
	Siberian	
	Domestic shorthair	[27]
MYH7 c.5647 G > A [E1883K]	No-breed	[30]
TNNT2:c.95-108 G > A	British shorthair	[32]
	British longhair	
	Ragdoll	
	Sphynx	
	Maine coon	
	Devon rex	
	**Maine coon**	[66]
ALMS1:c.7384 G > C [G2462R]	**Sphynx**	[32]
	Devon rex	
	Maine coon	
	Ragdoll	
	British short- or longhair	
	Two cats with no known breed	[33]
	American shorthair	[72]
	Exotic shorthair	
	Minuet	
	Munchkin	
	Scottish fold	

### 2.6. Effect of Homozygous and Heterozygous Mutation on HCM Phenotype

The occurrence of increased penetrance in those homozygous for a particular gene supports the theory of a toxic proteome as the cause of HCM in cats. In contrast, in humans, haploinsufficiency is the main cause [73]. In cats, homozygosity would be expected to lead to more mutated proteins, whose higher concentration leads to a more pronounced manifestation of the phenotype. Consistent with this, in Maine Coon (with MYBPC3 A31P), the septal and free wall thickness of the left ventricle and LA enlargement were noted only in homozygotes [74]. In the same study, there were no differences between heterozygotes and control cats [74]. In a plasma experimental study in MYBPC3 cats, LVPW in diastole in homozygotes had a diameter of 0.57 cm (0.49–0.63), in heterozygotes 0.52 cm (0.49–0.56) and in the wild type 0.46 cm (0.34–0.58) [8].

The survival time for MYBPC R818W cats for homozygotes is 5.65 years, the survival time for heterozygotes is 16.7 years, and the survival time for WT is 15.2 years [70]. In addition, homozygous cats were at a higher risk of cardiac death [70]. Homozygous cats had an LV wall of 5.37 mm, and heterozygous cats had an LV wall of 4.73 mm [3].

## 3. HCM-Related Gene Expression in Cats

An explanation of the true pathway of the HCM phenotype development in cats is still unavailable. Data show that pathological genetic variants have an extensive impact on the transcriptome of co-regulated genes [75]. However, we know more and more about the mechanistic remodeling processes that are present within cardiac tissue and are responsible for the development of HCM. The gene expression profile in the cardiac tissue of cats with HCM is different compared to healthy cats and also cats with inflammation throughout the body [76]. Regulation of the TGFβ1 and beta-estradiol TNFα pathways is reduced in lesional tissue compared to healthy tissue [77]. Increased expression of pro-inflammatory genes and extracellular matrix genes is noted [36,76,78]. Particularly interestingly, regional differences between the expression of pro-inflammatory genes (between atrial—LA and ventricular—LV) have been demonstrated, which has been termed differential gene expression (DEG). Atrial tissue has significantly higher gene expression compared to LV tissue (almost 1000 genes more abundantly expressed in the LA) [77]. This may explain why atrial tissue produces cytokines significantly more intensively [36,78,79]. Furthermore, markers of extracellular matrix activity are also more highly expressed in atria [36]. The secretory role has also been investigated in terms of atrial thrombus formation [76]. Higher expression of IL-6 has been shown when LA thrombus is present; however, coagulation and endothelial activation genes are not activated in the hearts of HCM cats [76]. In another study in cats with HCM, an increase in cytokines was accompanied by an increase in leptin mRNA; however, when a thrombus was present in the LA, leptin activity decreased [80]. Few genes are more strongly expressed in the LV. These include genes related to nicotinamide adenine nucleotide metabolism (ART5, ART1) and transcription factors (IRX4, IRX5, IRX3) [77]. A feline model of HCM was developed through the use of Drosophila [75]. Flies were modified for the presence of MYBPC3 mutations. The analyses of the ongoing cultures revealed 365 significantly different genes in the Wild-type, 146 of the A31P variant, and 139 of the R818W variant [75]. A total of 88 significantly altered genes were common to all three variants. The pathogenic variants (A31P and R818W) showed common changes in inflammatory response genes [75]. The increase in IL-1, IL-4, IL-6, TNF-α, TGF-β, MMP-9, MMP-13, TIMP-1, and TIMP-2 in cats with HCM is probably related to mediating the subsequent fibrosis of cardiac tissue and the increase in cardiac collagen content [36,79,81]. The increase in cytokines corresponds not only to the activation of the immune system due to the emergence of the damage-associated molecular pattern (DAMP) as a result of cardiomyocyte death and breakdown but also to the generalized inflammation of affected cats, which increases with HCM progression. Maintenance of the inflammatory and profibrotic environment has been attributed to increased macrophage activity [78]. The state of stimulation of cardiomyocytes and connective tissue cells (increased Ki-67 levels [82]) also deteriorates higher oxygen and substrate demands. Neovascularisation at sites of replacement fibrosis is noted; however, an ischaemic component is suspected in HCM [82]. Recent studies have demonstrated a reduction in coronary vascularisation (reduced vascular density) due to an expansion of extracellular matrix volume [82]. There was no increase in vascular epithelial growth factor activity in HCM, which was also confirmed in subsequent studies, and vascular endothelial activity was not crucial in the development of HCM [76]. However, this is not consistent with the neovascularisation shown in previous studies [78].

## 4. Other Mechanisms Involved in the Development of HCM

Epigenetics is increasingly influencing the understanding of the development of animal diseases. Epigenetics is closely linked to aging processes, the rate of which is accelerated in some disease entities [83,84]. Aging processes also provide an explanation for the processes controlling the development of HCM. Cellular aging is inevitable and contributes to dysfunctional cells. Natural Replicative Ageing mainly contributes to telomere shortening and exposure of chromosome ends—which is experienced by the cell as endogenous damage. External factors such as trauma and oxidative stress cause cells to rapidly accumulate DNA damage and undergo stress-induced aging [85]. Importantly, when cells undergo aging, they secrete numerous proteins as part of the ageing-associated secretory phenotype (SASP). The inflammatory profile that is seen in feline HCM, resulting from damage to sarcomere function [36,79,81] and mitochondrial dysfunction [86], may also be part of cardiomyocyte aging. Furthermore, SASP does not have a fixed composition and changes according to the phase of aging [87]. Originally composed of growth factors (TGF-β1 and TGF-β3), it then gains an inflammatory character (interleukins) [87]. The current influx of inflammatory cells, such as macrophages, may be a result of the chemokine stimulation that aging cells exert [78]. The mechanism responsible for the formation of SASP and the adoption of an aging phenotype by cells (enlargement and hyperexpression) is the mTOR regulatory pathway [88]. The mTOR system consists of two complexes: mTOR 1 and mTOR 2. mTOR integrates nutritional and metabolic signals through interactions with the insulin receptor; therefore, excessive mTOR activity can induce insulin resistance [89,90]. Both mTOR and IGF-1 signaling are simultaneously required for the stimulation of pathways involved in the proliferation of almost all cell types, so the relationship between their activity and sensitivity may be crucial for the bluntness of aging [91]. The association of HCM with aging processes is indicated by the relationship with IGF-1 levels, which is not only a regulator of cell proliferation but appears to influence the development of HCM [21]. Furthermore, the mTOR pathway has been implicated in cardiac remodeling and fibrosis [92,93,94]. In dogs with a myxomatous mitral valve, the mTOR pathway has been indicated to be involved in mitral leaflet remodeling [95]. The key role of this process is also confirmed by the response of rapamycin-treated HCM [96]. Rapamycin is a natural antifungal product of *Streptomyces hygroscopicus*. The rapamycin molecule binds to the multifunctional protein FKBP-12. The FKBP-rapamycin complex interacts with the FKBP-rapamycin-binding (FRB) domain, causing inhibition of the mTOR complex [92]. As a result, phosphorylation of 4E-BP1, cdks-p27, and P70s kinase is inhibited [92]. Cell proliferation and cell cycle arrest occur [92]. Rapamycin affects cell metabolism, immune response, autophagy, survival, proliferation, and migration, so rapamycin is successfully used by physicians, among others, to induce immunosuppression, e.g., after transplantation [97]. In recent laps, knowledge of the action of rapamycin has been developing rapidly, and its action is being tested on numerous animal models. Their translational nature is used in projects such as the Dog Aging Project, where the anti-aging property of rapamycin is being investigated [98,99,100]. In cats, similar projects include RAPACAT [96]. Subclinical HCM in cats treated with long-term rapamycin led to a reduction in LV diameter [96]. Reverse cardiac remodeling occurs as a result of rapamycin [101]. Unfortunately, data on the exact effect of rapamycin-mediated mTOR inhibition are not available, and the results are unfortunately not reproducible [101]. Some inflammatory pathways detected for HCM (ERK/MAPK [77], FOXO1 [102]) activated in cats in HCM are associated with mTOR signaling pathways and IGF-1 signaling [103,104]. Among the epigenetic mechanisms, DNA methylation should be singled out, the degree of which varies according to the developmental stage of the organism. It is responsible for cell proliferation and differentiation. Its fundamental importance has been reflected in pathological processes, as it has been demonstrated that it can be linked to remodeling and fibrosis processes in cardiac tissue [105]. Fibrosis of cardiac tissue facilitates the development of re-entry loops or isolation of ectopic sites and, consequently, the development of supraventricular and ventricular arrhythmias [106,107], which has also been indicated in HCM [108]. In feline HCM, arrhythmias are part of the phenotype that impairs prognosis and may have diagnostic potential [109]. Another known epigenetic process is the post-transcriptional regulation of protein synthesis. MicroRNAs (miRNAs) are regulators of mRNA expression and protein levels, which may be responsible for mechanistic processes in HCM development. In human HCM, a specific miRNA expression profile was detected in the MYBPC3 variant [110]. Increased expression of hsa-miR-320e, hsa-miR-486-3p, kshv-miR-K12-10b, hsa-miR-5700, hsa-miR-486-3p, hsa-miR-513a-3p, hsa-miR-1246, hsa-miR-381-3p, hsa-miR-376c-3p was distinguished in feline HCM. In contrast, reduced expression in feline HCM has been reported for kshv-miR-K12-5-5p and hsa-miR-3177-3p [111]. At this point, however, we do not know more in the context of miRNAs and HCM in cats.

## 5. Conclusions

Hypertrophic cardiomyopathy (HCM) in cats certainly has a strong genetic component, which is consistent with the knowledge of HCM in humans. Of the numerous genes that have candidate or suspect status, few have an indisputably proven role in the development of HCM in cats. The MYBPC3:c.91 G > C [A31P] and MYBPC3:c.2453 C > T [R818W] variants are breed-specific mutations (Maine Coon and Ragdoll, respectively) that show up familially, and the number of alleles in the genotype is associated with a more severe phenotype and worse prognosis. The other genes, except MYH7, are widely distributed in the cat population and segregate cats with and without HCM in only a few cases. Furthermore, the pathophysiological mechanism that leads from gene to phenotype remains a mystery despite the many discoveries that have been made. Several potential diagnostic and mechanistic targets, such as mTOR complex activity or aberrations in epigenetic profiles, have been discovered but are still being tested. Above all, we believe that genome-wide sequencing in large populations of specific mutation variants should be a leading research direction in the future. A second direction of research should become the processes of regulation of gene expression. A major limitation of HCM research in cats and a problem to be explained is the overwhelming majority of diagnosed HCM in non-breed cats, in which pathological variants are not detected. In addition, in the context of genetic testing, possible bias and potential small sample bias must be taken into account, as studies on cats are relatively small in number. We anticipate that in future years, their number will increase significantly due to their unique translational potential. Furthermore, a more in-depth knowledge of this disease in cats will contribute to improving it as a model for human HCM.
